# Benchmarking commercial depth sensors for intraoperative markerless registration in neurosurgery applications

**DOI:** 10.1007/s11548-025-03416-y

**Published:** 2025-05-23

**Authors:** Manuel Villa, Jaime Sancho, Gonzalo Rosa-Olmeda, Miguel Chavarrias, Eduardo Juarez, Cesar Sanz

**Affiliations:** CITSEM, Universidad Politécnica de Madrid, Madrid, 28031 Spain

**Keywords:** Depth sensors, Image registration, MRI, Computer-assisted intervention

## Abstract

**Purpose:**

This study proposes a generalization of markerless patient registration in image-guided neurosurgery based on depth information. The work builds on previous research to evaluate the performance of a range of commercial depth cameras and two different registration algorithms in this context.

**Methods:**

A multimodal experimental setup was used, testing five depth cameras in seven configurations. Fiducial registration error (FRE) and target registration error (TRE) metrics were calculated using iterative closest point (ICP) and deep global registration (DGR) algorithms. A phantom head model was used to simulate clinical conditions, with cameras positioned to capture the face and craniotomy regions.

**Results:**

The best-performing cameras, such as the D405 and Zed-M+, achieved TRE values as low as 2.36 ± 0.46 mm and 2.49 ± 0.35 mm, respectively, compared to manual registration that obtains a 1.37 mm error. Cameras equipped with texture projectors or enhanced depth refinement demonstrated improved performance. The proposed methodology effectively characterized the suitability of the camera for the registration tasks.

**Conclusion:**

This study validates an adaptable and reproducible framework to evaluate depth cameras in neurosurgical scenarios, highlighting D405 and Zed-M + as reliable options. Future work will focus on improving depth quality through hardware and algorithmic improvements. The experimental data and the accompanying code were made publicly available to ensure reproducibility.

## Introduction

Magnetic resonance imaging (MRI) is widely used in image-guided neurosurgery (IGN) due to its high spatial resolution, non-invasive nature, and ability to provide a good contrast response with soft tissue contrast [2, 12, 22, 37]. Although helpful as it is, this MRI has some limitations that have led to an increasing push to combine this technology with other medical techniques to enhance its usability. One such limitation is the location of the disease, which is a time-consuming task often performed manually that requires expert knowledge, leading to the research of automated solutions [4, 10, 23]. Another issue is the lack of an in situ response, as MRI is usually performed preoperatively and does not provide dynamic guidance during the procedure. This can be addressed by the generation of a multimodal system with preoperative MRI and other intraoperative image solutions [3, 8, 17].

Many dynamic imaging solutions are used to integrate preoperative and intraoperative images. These include computed tomography (CT) [17], positron emission tomography (PET) [41], ultrasound (US) [27], and hyperspectral imaging (HSI) [31, 38]. In all cases, the challenge facing these systems is the registration of preoperative MRI with intraoperative images obtained during surgery. This process is not straightforward and often relies on semiautomatic methods that depend on manually selecting the corresponding points on both the MRI and the intraoperative image. However, this manual selection is prone to variability between operators and is very time-consuming [5].

This work is based on a previous work, HyperMRI [45], in which a novel multimodal augmented reality (AR) methodology was proposed for the registration of MRI and HSI. The methodology employs an Azure Kinect DK RGBD camera and an Optitrack tracking system to dynamically combine the information provided by preoperative MRI and the classification map generated from a HS camera in a fully automatic way. In this process, registration between the preoperative and intraoperative environments is carried out by aligning the preoperative 3D volume with the patient’s face during surgery. This step is crucial to the methodology, as the quality of subsequent registration is heavily dependent on both the quality of the captured 3D face and the algorithm used to align it with the preoperative volume.

Building on this approach, the present work aims to generalize the methodology to any depth camera by changing the camera-tracking calibration, allowing to register and track any RGBD camera (the previous method was restricted to IR-based cameras). Using the novel methodology, we compared five different RGBD cameras with various depth acquisition technologies and two distinct algorithms for the registration process. The goal is to evaluate how the performance of each camera impacts the quality of the registration and to evaluate the effectiveness of each algorithm in aligning the intraoperative data with the preoperative 3D volume. By conducting this comparison, we identify the most suitable combination of camera and algorithm to improve the accuracy and performance of the registration in an image-guided neurosurgical context.

The findings indicate that using this methodology, two camera-algorithm pairs stand out from the others, achieving a target position error (TRE) of 2.36 mm for the Intel D405 camera and 2.49 mm for the Zed-M+, respectively. These values significantly improve upon the approximately 4 mm error reported in previous work with the Microsoft Kinect Azure camera. Furthermore, the results achieved are comparable to the manual registration method, which yielded an error of 1.37 mm, highlighting the effectiveness of the automatic method.

## Related works

The main problem with image registration in the medical field is the lack of precise registration. Dynamic AR multimodal systems generally manage this by employing depth sensors and real-time tracking devices.

**Fig. 1:**
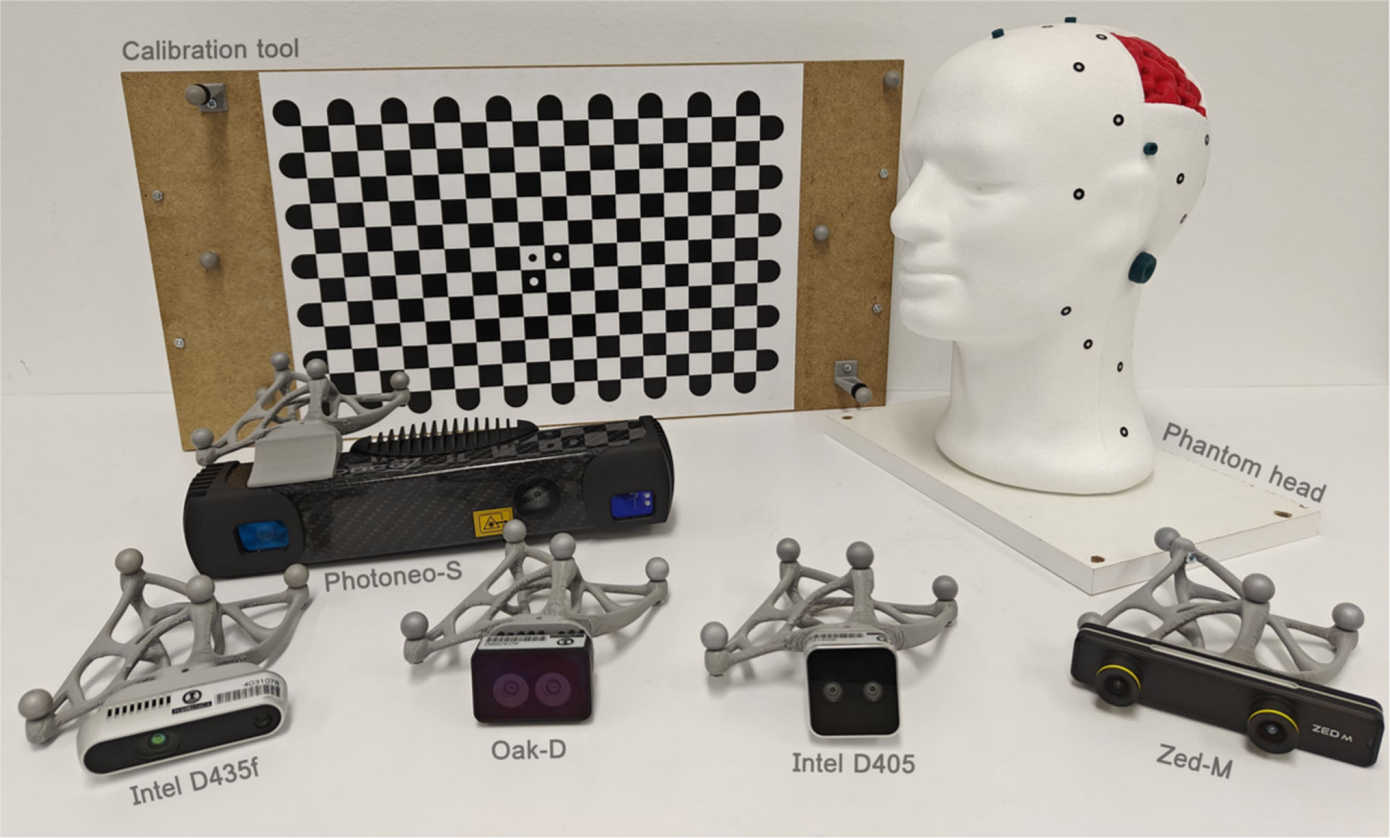
Experimental setup. Materials used in the experiments: cameras (bottom) with their respective tracking objects attached, the calibration tool (checkerboard and tracking markers, upper left), and the phantom head and brain (upper right)

An important trend in multimodal AR systems involves head-mounted displays (HMDs) with depth cameras, such as HoloLens 2. Equipped with a ToF sensor and IR cameras, the HoloLens 2 enables tracking through reflective markers without the need of an external tracking system. Several studies [16, 25, 32] have successfully used HoloLens 2 in various medical settings, reporting millimeter-level accuracy. Alternatively, some researchers use standalone commercial depth cameras [18] (Intel RealSense D415 [19]), [28] (Zed mini [42]), [47] (Intel D415), [24] (stereo HikVision MV-CA023-10GM) along with a tracking system to achieve an AR system with a similar accuracy. In these works, the AR system is employed to assist spine surgeries, iliac crest transplant, or oral and maxillofacial surgeries, enhancing surgeons’ efficacy and precision.

AR systems have also been employed in neurosurgery, aiding in preoperative planning, intraoperative navigation, and enhancing surgical precision. One example is [13], where the authors utilize an AR system to enhance navigation through the brain’s internal structures (including tumor), which were previously segmented from a preoperative MRI. Similarly, in [29], the authors developed an augmented reality (AR) system that integrates preoperative CT scans with real-time RGB visualization, using fiducial markers and deep learning for accurate registration. With this system, they aim to enhance surgical navigation by leveraging preoperative data for improved precision and guidance.

All these studies share a common approach; they register patient’s volume information acquired preoperatively with a real-time depth camera. This process heavily relies on the depth information provided by the depth cameras and the registration algorithm, as shown in works such as [6], for depth cameras, and [9] for registration algorithms. Extending this approach, our work compares several commercial depth cameras and registration algorithms in the context of surgical applications to assess their performance in multimodal augmented reality systems for surgeries.

## Materials and methods

This study focuses on comparing five commercially available depth cameras under controlled conditions, assessing their performance in aligning point-cloud data to preoperative synthetic MRI volumes. In addition, a neural network-based approach was employed and evaluated along with the widely used iterative closest point (ICP) algorithm to determine the optimal registration strategy. The following subsections detail the equipment, experimental setup, algorithms, and evaluation metrics used in this study.

### Experimental setup

The five depth cameras used in this study are: (i) Intel RealSense D405 [21], (ii) Intel RealSense D435f [20], (iii) ZED-M by StereoLabs [42], (iv) OAK-D Short Range by Luxonis [30], and (v) MotionCam-3D S by Photoneo [36], as illustrated in Figure 1. These cameras are a representative sample of commercial options that are widely accessible, with their main features summarized in Table 1.

Three different depth-camera technologies were employed to identify the most suitable option for this use case. The experimental setup includes cameras with short- (D405, Oak-D and Photoneo-S) and long-range (Zed-M, and D435f) depth capabilities. These cameras feature varying resolutions, including 720p HD for Intel cameras, Full HD for Zed-M, and specific resolutions of 1280$\times$800 and 1120$\times$800 for Oak-D and Photoneo-S, respectively. In addition, projectors were used to enhance depth measurements in two different configurations. The D435f camera features an infrared projector with a fixed semi-random dot texture, while the Photoneo-S camera uses an infrared projector that functions as both a time-of-flight camera and a structured light system for depth sensing. Furthermore, depth refinement is used in the Zed-M camera, which utilizes a neural network to improve accuracy and smooth depth data.

**Table 1:** Cameras specifications

**Camera**	**Depth resolution**	**Depth technology**	**Depth range**
**D405**	$1280\times 720$	IR stereoscopic	0.07 - 0.5 m
**D435f**	$1280\times 720$	IR stereoscopic + projector	0.3 - 3 m
**Zed-M**	$1920\times 1080$	RGB stereoscopic + NN	0.1 - 9 m
**Oak-D**	$1280\times 800$	RGB stereoscopic	0.3 - 1 m
**Photoneo-S**	$1120\times 800$	Parallel structured light	0.33 - 0.55 m

The purpose of the experiments is to evaluate the effectiveness of each camera in the generalized methodology proposed in this work. For additional options, such as the dot projector in the D435f camera and the neural network refinement in the Zed-M camera, the cameras will be tested in both configurations, using the enhancements and without them, to assess their impact on depth measurement performance.

The experimental setup was designed to evaluate the performance of the selected depth cameras under controlled conditions, specifically for the task of registering a patient’s MRI with real-time data in the operating room. To simulate this scenario, in addition to the depth cameras, a commercial tracking system was used to locate the cameras in the 3D space. The tracking system consisted of four Optitrack Flex 3 cameras [35], coupled with Motive software, which was responsible for retrieving the positions of rigid bodies within the setup.

To simulate the conditions of MRI registration, a mannequin head phantom was used. Rather than recreating a complete patient head, an expanded polystyrene (EPS) head served as the base, ensuring realistic facial features such as a bigonial width of 118 mm, an alar base of 35 mm, and a nose length of 50 mm, among others [15]. A brain segment, 3D-printed from a real MRI scan, was integrated into the EPS head, as shown in Figure 1. This allowed a visual assessment of the alignment between the brain MRI and the head segment. The point-cloud data were acquired using an EinScan Pro 2X 3D scanner (Shining 3D) [40] and processed with 3D-Slicer software [1] to generate synthetic MRI slices. The head surface was converted into volumetric data in 3D-Slicer by adjusting the scan spacing to match real-world MRI, as resolution variations can impact registration accuracy metrics.

Using the phantom head and the tracking system, the same conditions are ensured for the five cameras. The registration procedure used in this study follows the approach defined in the HyperMRI work [45]. Specifically, the information used for patient registration includes the face, which is extracted from an RGB image and reprojected onto the depth camera. The point cloud of the patient’s face is then captured by the depth camera and aligned with the corresponding point cloud derived from the synthetic MRI data (in this work, the phantom head). For this alignment, a neural network designed for point-cloud registration, known as deep global registration (DGR) [11], is used in conjunction with the traditional iterative closest point (ICP) algorithm [26], to test registration performance.

### Dataset acquisition

**Fig. 2:**
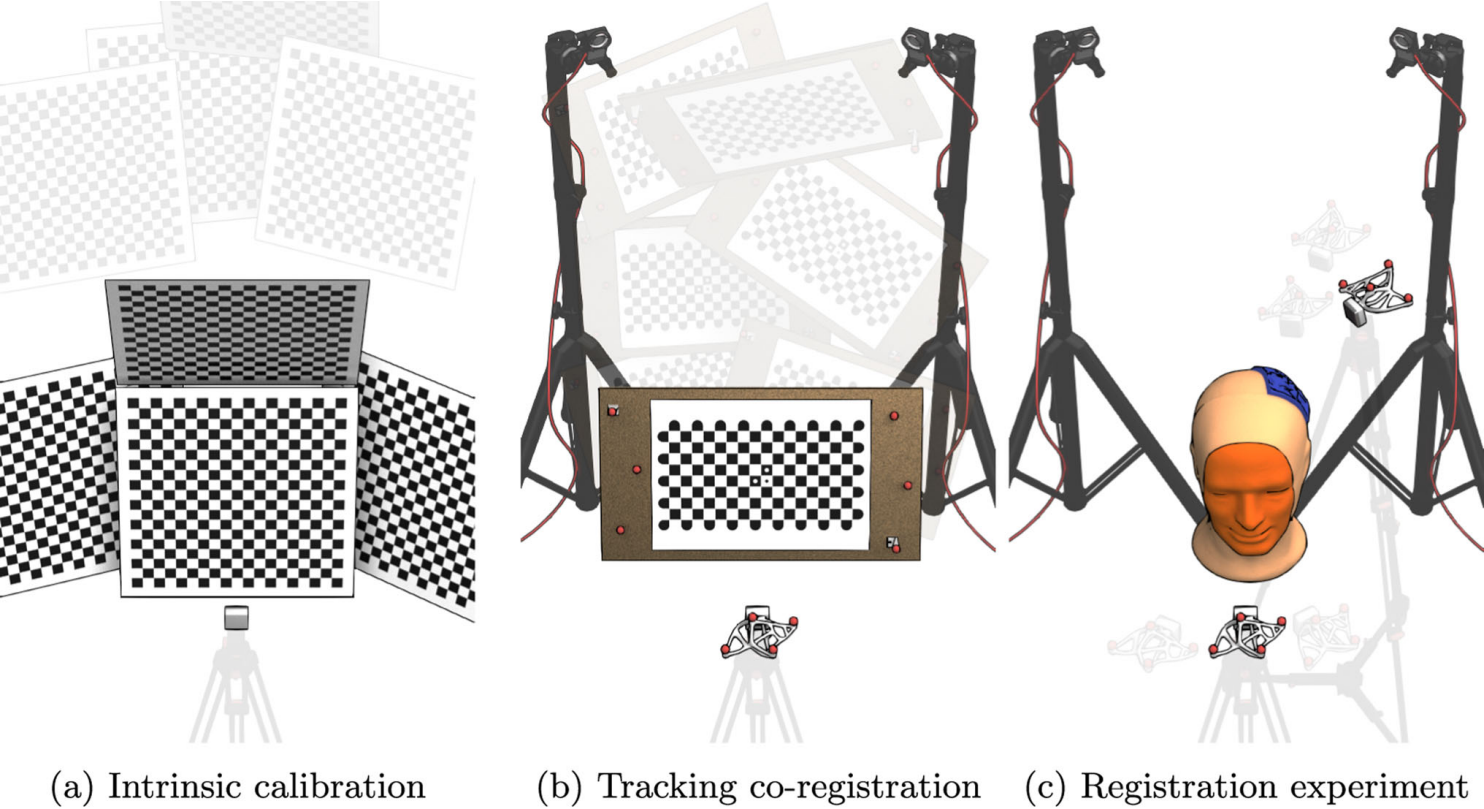
Dataset acquisition methodology. For each camera, the same procedure was followed: (a) calibration of intrinsic parameters using a checkerboard at two depth planes, (b) establishing the relationship between the camera’s optical center and the tracking system’s coordinates with the calibration proposed, and (c) positioning the camera in two zones around the patient phantom-one for capturing the face and the other for focusing on the exposed brain region. This process was repeated 10 times to evaluate camera accuracy

To ensure a fair comparison between the cameras, a dataset was acquired using a methodology developed in this work and made publicly available as open-source^1^ as well as the code needed to capture, calibrate and register the information^2^. The following steps were performed for each camera: (a) characterization of intrinsic parameters, (b) co-registration within the tracking system, (c) registration and testing captures, as depicted in Figure 2. These stages are explained in more detail in the following subsections.

#### Intrinsic camera calibration

The intrinsic calibration is performed using the DLR CalLab framework (German Aerospace Center) [43]. This calibration includes the projection matrix (K matrix) and distortion parameters for the main RGB camera used in the system, which needs to be aligned to the depth information. Depending on the camera, the RGBD image is generated in a different position: the left camera (Zed-M, Oak-D), the virtual position of the center camera (D405), or the real RGB camera (D435f, Photoneo-S).

The calibration process is performed by extracting eight captures from a Radon checkerboard [34] in different positions, as shown in Figure 2a. Four of the positions correspond to the checkerboard tilted at four different orientations, while the remaining four captures to the same orientations but with the checkerboard placed at a different depth plane. These depth planes are determined based on each camera’s field of view and resolution, ensuring a consistent approach across all devices by covering at least one edge of the sensor in each capture to achieve a robust characterization of the radial distortion parameters.

#### Co-registration within the tracking system

To perform co-registration within the tracking system, each camera was mounted with a specifically designed tracking object to ensure precise localization. Initially, reflective markers were attached directly to the camera bodies. However, due to the camera’s limited surface area, this approach resulted in insufficient marker dispersion, making it difficult to accurately determine the cameras’ rotation within the Optitrack system. To address this issue, the tracking objects were redesigned to provide better marker distribution and to enable precise tracking of both the camera location and orientation, as illustrated in Figure 1.

**Fig. 3:**
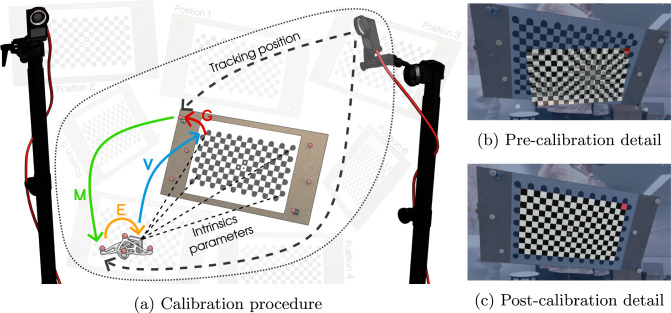
Co-registration of cameras within the tracking system. (a) Schematic representation of the calibration procedure for each camera and the transformation details. (b) Real-world capture of the D405 camera in the augmented reality interface developed for the calibration procedure. (c) Calibration result showing perfect alignment between the virtual and physical checkerboard

Once the cameras could be reliably tracked by Optitrack, their captured images needed to be co-registered to the Optitrack coordinate system, specifically aligning the optical center of each camera to its corresponding tracking object (transformation E in Figure 3a). The co-registration approach, originally presented in [45], was designed for IR-based depth cameras, such as the Microsoft Kinect Azure [33]. Unlike the original method, which relied on a calibration tool with three reflective markers, this procedure has been adapted to work with any type of camera within the tracking area. This adaptation enables the integration of various imaging technologies, including hyperspectral cameras, thermal cameras, or any camera with clinical relevance. Indeed, camera resolution and checkerboard size factors will influence the precision.

The calibration process involves capturing multiple images of a checkerboard while simultaneously retrieving the tracking positions of both the camera and the checkerboard. To ensure that the checkerboard can be tracked like the cameras, reflective markers are attached to it at varying heights for better tracking performance. Using the positions provided by the tracking system of the two rigid bodies (camera and checkboard), a relative transformation between them is calculated (transformation M in Figure 3a). The relative position of the optical center and the checkerboard is estimated using Zhang’s calibration method [7], implemented in OpenCV (transformation V in Figure 3a). Combining this information, the relative transformation between the rigid body of the camera and its optical center (E) and between the checkerboard’s origin and its rigid body (G) can be calculated through an optimization procedure, as described in [14].

Both transformations, E and G, must be estimated simultaneously. The relationship between the variables is expressed in Equation 1. To achieve this, an optimization procedure is conducted in which the standard deviation of the checkerboard points is minimized by transforming them according to the equation. Optimization is performed using the L-BFGS-B method of the Scipy library [39].

1 $$\begin{aligned} G = (M\cdot E\cdot V)^{-1} \end{aligned}$$

The calibration procedure is interactive, facilitated by an AR interface developed in Python using VTK [46]. This interface allows the optimization procedure to be relaunched and updated in real-time for each new capture. This interactive approach not only calibrates the cameras but also enables the immediate evaluation of calibration quality. By visually inspecting the overlap between the actual checkerboard and the virtual checkerboard, positioned according to the transformations, users can assess the precision of the calibration in situ, as depicted in Figure 3b and Figure 3c.

#### Register experiment

**Fig. 4:**
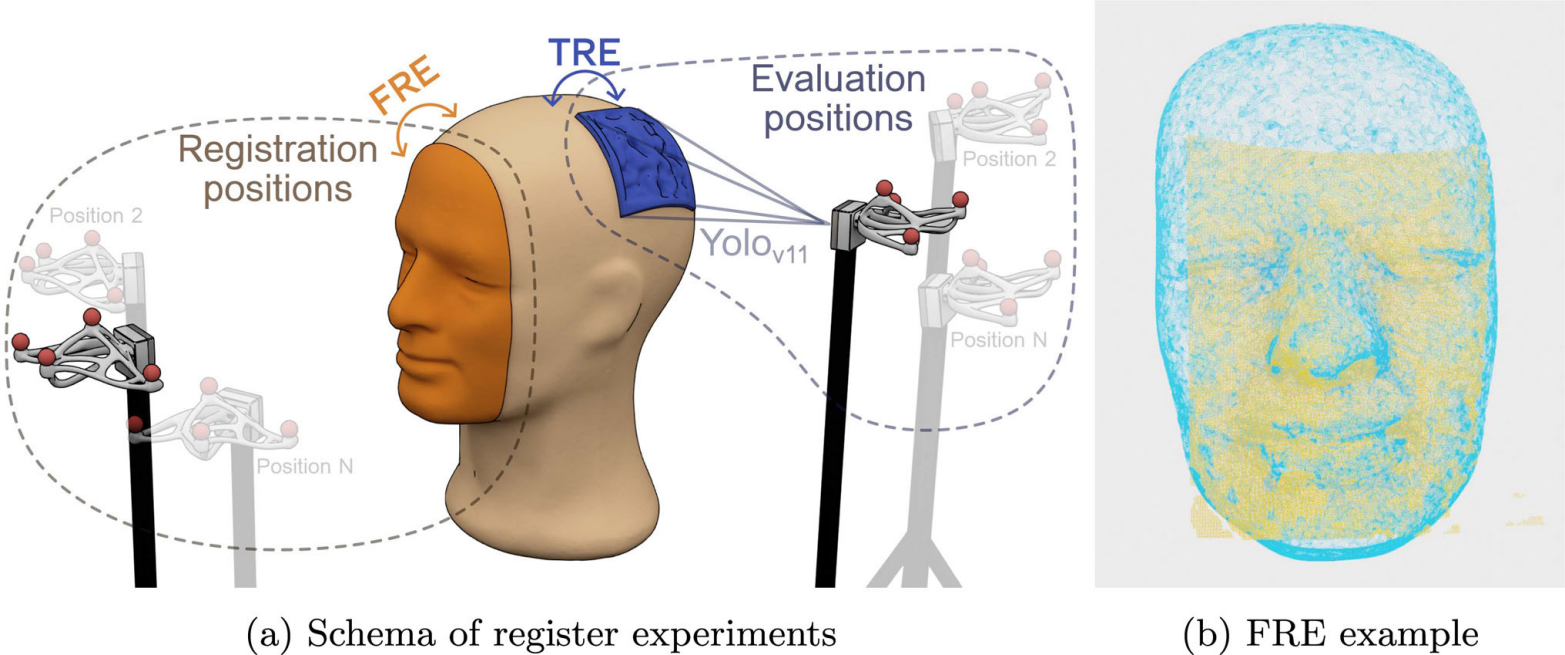
Registration experiment. (a) Schematic representation of the registration experiment conducted with each camera, illustrating the information utilized to compute each metric (lighter-colored cameras represent the various positions). (b) Example of registration results using the D405 camera at the registration position, showing the overlap between the camera point cloud (orange) and the MR-derived facial surface (blue)

To evaluate the performance of the cameras, the experimental procedure involved placing them in two distinct locations relative to the patient phantom. These positions were used to compute the registration metrics for camera comparison, simulating realistic conditions in an operating theater. This setup reflects the clinical scenario where patient registration must be completed before craniotomy, after which the patient’s face is covered, necessitating camera relocation to visualize the surface of the brain, while maintaining an approximate distance of 40 cm to avoid the sterile surgical area. The parts of the phantom head used to extract each metric are detailed in Figure 4.

In the first position, called the registration position, five captures were taken at each of ten different locations to register the patient and calculate the fiducial registration error (FRE). In the second position, referred to as the testing position, the same procedure and number of images have been followed to compute the target registration error (TRE) as shown in Figure 4a. This dual-position approach ensures a complete characterization of each camera.

The configuration of the registration algorithms used in this experiment is as follows: ICP is based on the Open3D implementation of point-to-point ICP, incorporating global registration with RANSAC for robust alignment. DGR was utilized with the pre-trained weights, with the voxel size adjusted from the original 5 cm to 5 mm to better suit the specific requirements of this use case.

For the FRE calculation, only the patient’s face was used, as this constitutes the information used by the registration algorithms to compute the transformation, following the procedure described in [45]. For the TRE calculation, distinct points that were not involved in the registration process were used. Specifically, a YOLOv11 network [44] was fine-tuned to automatically detect the craniotomy site of the patient (blue in Figure 4b), allowing the TRE metric to be calculated exclusively within the region of interest. This approach minimizes the influence of extraneous elements, such as background information, and ensures that the evaluation focuses solely on the relevant anatomical area. The bounding boxes generated by YOLOv11 were manually reviewed to confirm accurate detection of the ROI and to maintain a fair comparison across different cameras by ensuring a consistent margin around the region of interest.

To establish a baseline comparison, a manual registration of the phantom patient was performed by an experienced user and a non-trained user. This baseline serves as a reference for evaluating the performance of the automated registration methods and the accuracy achieved by each camera. Manual registration was performed using a fiducial marker approach, with markers specifically designed to ensure precise placement of the probe during the registration process. This setup represents an ideal scenario where the user has sufficient experience to perform an accurate registration. In real-world scenarios, landmarks are sometimes manually selected, which can introduce greater human bias. The process utilized the Micron Series Digitizing Probe (Optitrack) and an interactive interface developed in Python using VTK to compute the landmark transformations necessary for registration.

For this manual registration, the phantom head was equipped with eight rigidly attached fiducial markers, which were also manually identified in the MR slices using the 3D-Slicer software [1]. Half of these markers were used for registration (FRE), while the remaining markers were used for evaluation (TRE). This dual approach ensures a robust comparison of manual and automated registration methods across different cameras.

## Experimental results

This section presents and discusses the results obtained using the proposed methodology for the five commercial depth cameras listed in Table 1.

### Calibration

The results of the calibration process for each camera are included in Table 2. These results comprise both the intrinsic camera calibration and the co-registration with the external tracking system.

The intrinsic camera calibration error represents the projection error for each camera in pixels, calculated using the intrinsic matrix and the calibration captures. The results presented below are the direct output of the calibration software. As can be observed, the error for every camera is sub-pixel, achieving the best results for the Intel D405 and Photoneo-S.

In contrast, the co-registration error is calculated by projecting the points of the virtual checkerboard onto the image captured by the RGB camera (which is already aligned with the depth camera), using the transformation detailed in Section 3.2.2. This error represents the average Euclidean distance between the projected points in 2D and the corresponding captured points. Notably, the co-registration error is consistently sub-pixel. Furthermore, the number of captures varies as the process can be stopped once the error reaches an acceptably low level, below a quarter of a pixel considered a reliable stopping criterion.

**Table 2:** Results of the calibration of each camera. It includes the intrinsic camera projection and the co-registration error from the camera to the tracking system

	**Camera intrinsics**		**Co-registration**		
	**Proj. Error (px)**	**Captures**	**Proj. Error (px)**	**Captures**	**Resolution**
**D405**	0.38	8	0.21 ± 0.10	15	$1280 \times 720$
**D435f**	0.67	8	0.16 ± 0.08	15	$1280 \times 720$
**Zed-M**	0.57	8	0.21 ± 0.09	15	$1920 \times 1080$
**Oak-D**	0.68	8	0.10 ± 0.02	9	$1280 \times 800$
**Photoneo-S**	0.30	8	0.08 ± 0.04	13	$1120 \times 800$

### Registration

The comparison of cameras for the registration task, as described in Section 3.2.3, involved two experiments: one evaluating the performance at the registration position (measuring FRE) and another at independent points (quantifying TRE). Five cameras in seven configurations were analyzed using two registration algorithms, ICP and DGR. Also, manual register based on landmarks has been realized by the author and a non-expertized user, utilizing predefined reference points to ensure high registration quality and characterize the minimum achievable error with this approach. Under standard conditions, these points are not clearly defined, which can lead to higher errors due to human bias. The results are summarized in Table 3.

**Table 3:** Registration error in RMSE [mm] for each camera with different registration algorithms and manual register from different operators. Captures with less than 95% overlap were excluded due to unreliable results

	**FRE**		**TRE**	
	**ICP**	**DGR**	**ICP**	**DGR**
**D405**	1.60 ± 0.78	1.52 ± 0.45	2.36 ± 0.46	3.17 ± 0.29
**D435f**	1.63 ± 0.76	1.52 ± 0.41	3.83 ± 0.69	3.97 ± 0.48
**D435f w/o proy.**	Overlay < 95%	Overlay < 95%	-	-
**Zed-M**	3.24 ± 0.30	3.14 ± 0.20	Overlay < 95%	Overlay < 95%
**Zed-M +**	2.47 ± 0.61	2.07 ± 0.21	2.49 ± 0.35	3.26 ± 0.23
**Oak-D**	Overlay < 95%	Overlay < 95%	-	-
**Photoneo-S**	1.30 ± 0.95	1.65 ± 1.00	2.99 ± 1.50	3.15 ± 0.58
	**Author**	**User**	**Author**	**User**
**Manual register**	0.48 ± 0.07	0.68 ± 0.14	1.28 ± 0.26	1.41 ± 0.57
**Overall**	0.64 ± 0.22		1.37 ± 0.22	

For each camera, a transformation was computed at 10 distinct positions within the registration setup, maintaining a mean depth range of $44.22\pm 11.22$ cm across all cameras and positions. At each position, five captures were collected and a mean transformation was derived to map the patient’s MR data to the tracking system’s coordinate frame. To evaluate the TRE, additional captures were taken at 10 evaluation positions, with five captures per position, maintaining a mean depth range of $45.38\pm 14.94$ cm across all cameras and positions. These evaluation positions focused exclusively on the brain region of interest, specifically the craniotomy site, by cropping the point clouds using the bounding box of the brain region.

The point clouds from the cameras were compared with the registered MR data, and the FRE and TRE metrics were computed using the Open3D library. Initially, an overlap analysis was performed between the point clouds, with only overlapping points considered for the distance calculation. Registrations with less than 95% overlap were excluded as they yielded unreliable results, classifying them as failed registrations.

The results indicate a notable variability in camera performance. Photoneo-S achieved the lowest FRE with ICP (1.30 ± 0.95 mm), followed closely by D405 (1.60 ± 0.78 mm) and D435f (1.63 ± 0.76 mm). Zed-M + also showed strong performance, particularly when using DGR (2.07 ± 0.21 mm). In contrast, cameras such as the Oak-D and D435f without projector failed to meet the minimum overlap requirement, highlighting limitations in their ability to reliably align patient data.

For TRE, the D405 demonstrated consistent performance in both algorithms, with values of 2.36 ± 0.46 mm (ICP) and 3.17 ± 0.29 mm (DGR). Similarly, the Photoneo-S achieved 2.99 ± 1.50 mm (ICP) and 3.15 ± 0.58 mm (DGR). Both cameras provided high-quality results in an automatic way, with deviations of around 1 mm compared to manual registration. Although some configurations, such as Zed-M+, showed slightly higher TRE values, DGR generally exhibited greater variability than ICP, potentially indicating differences in the robustness of the algorithms.

These findings emphasize the importance of selecting appropriate camera configurations and algorithms for precise intraoperative registration. Photoneo-S and D405 emerged as the most reliable options, consistently achieving high accuracy across both FRE and TRE. Moreover, the results highlight the significant impact of depth quality enhancements on registration performance when cameras are complemented with a texture projector and/or a refinement neural network. For example, the D435f without the projector failed to achieve sufficient overlap to accurately register the patient. However, when the projector was enabled, the camera’s performance improved substantially, yielding results comparable to other high-performing cameras.

This underscores the critical role of supplementary features, such as projectors and post-processing techniques, in enhancing depth data quality. However, the inability of certain cameras to meet the overlap threshold illustrates the need for thorough validation of camera configurations in specific clinical setups to ensure robust and reliable registration performance.

## Conclusion and future work

This study presents a comprehensive evaluation of a significant range of commercial depth cameras for the registration task, showcasing significant contributions to the field. First, the methodology was generalized to support a variety of depth cameras beyond the Microsoft Kinect used in HyperMRI [45], enabling broader applicability. A dataset capturing all experimental data was made publicly available, along with the accompanying codebase, ensuring reproducibility and further research. Key innovations include an interactive augmented reality interface for camera calibration and a landmark registration interface.

The results highlight a significant improvement in TRE compared to HyperMRI, where a TRE of approximately 4 mm was achieved. In this study, the best-performing cameras, the D405 and Zed-M+, achieved a TRE as low as 2.36 and 2.49 mm, respectively, demonstrating their reliability for registration tasks when compared to the manual registration method (with predefined reference points), which yielded a 1.37 mm error. Furthermore, this paper validates the proposed methodology as an effective framework for characterizing depth cameras in clinical registration scenarios.

The compact size and affordability of these cameras make them practical for integration into real operating rooms with minimal disruption to surgical procedures. Implementing this type of automated registration could significantly reduce registration time compared to manual methods, which are highly dependent on the expertise of the clinician. Although the achieved registration accuracy closely matches that of an ideal manual scenario using predefined reference points, real-world conditions introduce human bias and variable constraints, potentially increasing error. This underscores the value of automatic registration methods in improving consistency and reliability in clinical settings.

Future work will focus on several directions to enhance the findings of this study. Adding a texture projector or depth refinement to the best-performing cameras, such as the Zed-M+ and D405, could improve depth quality and registration accuracy, while also exploring different depth ranges to assess their impact on performance. Further improvements in the tracking system could help reduce TRE, enabling even greater precision in clinical applications. Additionally, fine-tuning the DGR network with point clouds specific to this use case, rather than generic room-based datasets, could enhance its performance and robustness in similar medical scenarios. To further validate this methodology, future studies will aim to test it on real patients.

## Footnotes

1 Public available in Zenodo: https://doi.org/10.5281/zenodo.14627320

2 https://gitlab.citsem.upm.es/public-projects/immersive-imaging/benchmarking-commercial-depth-sensors.git
